# New Thrombolytic Infusion Application of Dissolving Renal Artery Embolic Thrombosis: Low-Dose Slow-Infusion Thrombolytic Therapy

**DOI:** 10.1155/2018/1609025

**Published:** 2018-05-02

**Authors:** Ahmet Karakurt

**Affiliations:** Department of Cardiology, Faculty of Medicine, Kafkas University, Kars, Turkey

## Abstract

Renal artery thromboembolism (RATE) is an uncommon complication of renal arteries from heart chamber. Although there is no treatment protocol prescribed with guidelines, thrombolytic agents such as rt-PA are frequently used. Unfortunately, current thrombolytic agent application protocol in treatment for the RATE is used in acute myocardial infarction or acute pulmonary embolism. In this protocol, 0.9–1.0% cerebral and 4–13% noncerebral hemorrhages are seen. In contrast to this protocol, we aimed to present a case of RATE, in which we applied low-dose, slow-infusion thrombolytic therapy, and we have not observed any complication such as cerebral and noncerebral hemorrhage.

## 1. Introduction

Although renal artery thromboembolism (RATE) is rarely observed, it is a serious condition that can result in renal infarction. This case is very difficult for physicians to treat. Anticoagulation and thrombolytic and invasive procedure (thrombus aspiration and/or ballooning/stenting) are applied in the treatment of that patient group. If the anticoagulant therapy is administered alone, thrombosis cannot be completely lysed. If the invasive intervention is applied, complete opening cannot be achieved in the occluded region due to multiple thromboembolisms. Moreover, it may lead to slow-flow phenomenon or complete stoppage (no-flow phenomena) in the distal region due to micro- or macroembolism. If a short-durational and high-dose thrombolytic agent (100 mg, for 2 hours), such as recombinant tissue plasminogen activator (rt-PA), is applied, the risk of cerebral or noncerebral hemorrhage may increase [1, 2]. In this study, we aimed to present a case of complete thrombolysis with low-dose slow-infusion thrombolytic therapy (LDSITT) without any complication in a patient with >80% occlusion of the branches of left renal artery due to thromboembolism.

## 2. Case Report

A 65-year-old male with diagnosed RATE was referred to our Emergency Department complaining of a 1-day history of left upper quadrant and left flank pain from the external center. Complaints occurred after palpitation lasting 45 minutes. The pain started from the left back region as if a knife struck. The intensity of pain increased over time; there was no change in the severity of pain due to the body position changes. He denied recent fever, trauma, chronic atrial fibrillation cardiomyopathy, coagulation disorder, prior history of thromboembolic disease, dysuria, hematuria, or change in bowel pattern. He had been smoking 20 cigarettes per day for 17 years and did not consume alcohol. He had a past medical history of a stent placed in a coronary artery due to acute coronary syndrome three months ago. He did not use prescribed aspirin, metoprolol, clopidogrel, and atorvastatin agents recommended for acute coronary syndrome and stenting.

On physical examination, blood pressure, pulse, and respiratory rates were 144/80 mmHg, 69/min, and 22/min, respectively. Arterial blood oxygen saturation and fever were also 90% and 36.2°C, respectively. The electrocardiogram showed normal sinus rhythm. Cardiac examination revealed a fourth heart sound in the apical focus.

Biochemical tests revealed that fasting blood glucose level (182, 70–115 mg/dl), white blood cell (11.9, 3.7–10.4 K/uL), neutrophil count (9.63, 1.8–7.8 K/uL), and C-reactive protein level (18.92, 0–0.5 mg/dl) had increased. There was neutrophil dominance. Platelet count (111, 150–450 K/uL), calcium (7.5, 8.8–10.2 mg/dl), and uric acid levels (2.5, 3.4–7 mg/dl) had decreased. Troponin-I (0.01, <0.3 ng/mL) and creatine kinase-MB isoenzyme levels (51.9, 0–24 U/L) and glomerular filtration rate (70.07) were normal. By urine analysis, +2 glucose, +2 proteinuria, and +2 bloods were detected. Other urine parameters were normal.

A transthoracic echocardiogram revealed hypokinesis of the anterior and anterolateral wall and left ventricular ejection fraction was calculated as 58% by using the modified Simpson technique. There was no thrombus formation in the heart chamber and wall. Lower extremity venous Doppler ultrasonography examination was normal.

**Figure 1:**
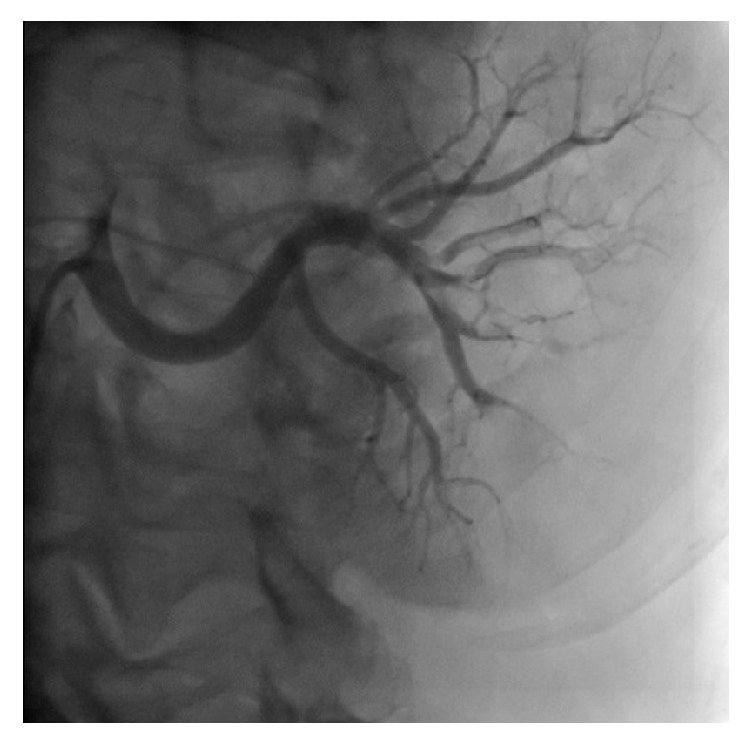
First renal artery angiography showing the multiple thromboembolic materials in the left renal artery branches.

The coronary angiography revealed a stent in the left coronary artery and it was open. Other coroner arteries were normal. The renal artery angiography revealed that there were multiple vessel diseases with >80% occlusion with thrombus in right renal artery branches.  Figure 1 and Video 1 show the multiple thrombosis in the right renal artery branches.

Depending on clinical, electrocardiographic, biochemical, and angiographic evidence, the patient was diagnosed with the thromboembolic occlusion in the left renal artery branches developing due to unknown origin.

We decided to use LDSITT for the occluded renal artery branches because the patient was not eligible for percutaneous therapy. Antiplatelet agents (aspirin 100 mg and clopidogrel 800 mg) were given prior to invasive process as a premedication from the referral hospital. Neither unfractionated heparin (UFH) (IV bolus end continue infusion) nor low-molecular-weight heparin (LMWH) was given prior to the LDSITT.

### 2.1. LDSITT Administration

**Figure 2:**
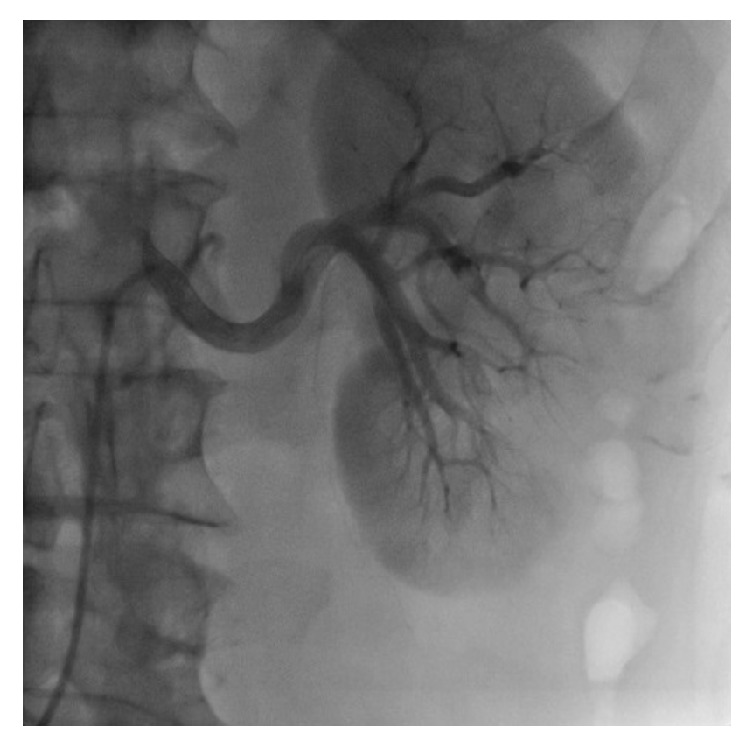
Second renal artery angiography showing residual thromboembolic materials in the left renal artery branches.

**Figure 3:**
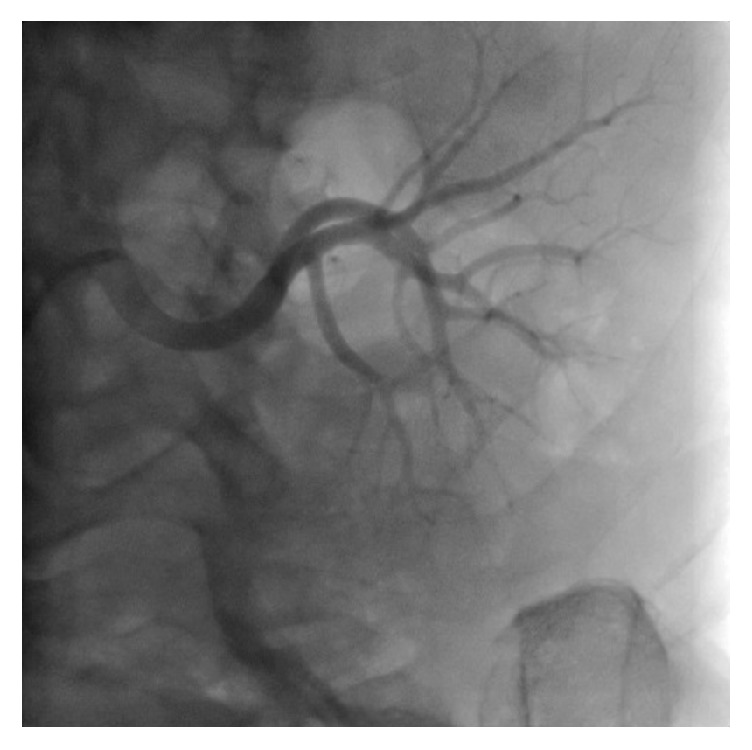
Final renal angiography showing completely lysed thromboembolic materials in the left renal artery branches.

The patient was given an intravenous infusion of 24 ml rt-PA (low dose) in 100 ml normal saline in 48 h (slow infusion time) twice. Following the second dose of fibrinolytic therapy, coronary angiography showed partial regression of the thrombus, leaving a residual thrombus.  Figure 2 and Video 2 show residual thrombosis in the right renal artery branches. Therefore, a third dose of 24 ml rt-PA was administered to the patient to completely lyse the thrombus. The third control renal angiography revealed complete lysis of the thrombus with no residue following the third dose of thrombolytic administration. In total, 72 mg rt-PA was administered.  Figure 3 and Video 3 show that multiple thrombosis is completely solved.

Although the thrombus was completely lysed after a total of three LDSITT, no complication developed other than minimal ecchymosis at the injection site. On the fifth day of admission, the patient was discharged with the prescription of aspirin 100 mg 1 × 1 and clopidogrel 75 mg 2 × 1 and the recommendation of Internal Medicine Department's outpatient control for diabetes mellitus regulation.

## 3. Discussion

The true incidence of renal infarction due to renal artery thromboembolism is unknown. Its incidence is reported as 1.4% in an autopsy series of 14411 cases and 0.02/1000 at another series [3, 4]. Depending on the rare occurrence of renal artery thromboembolism due to its rareness and its being less known, a correct diagnosis and a correct treatment are often delayed. If spontaneous renal artery thrombosis is not diagnosed early and is not treated properly, it results in renal insufficiency due to renal infarction.

RATE treatments are antiplatelet, anticoagulant, and/or thrombolytic treatment and/or stent implantation with/without appropriate thrombus aspiration procedures. Thrombolytic therapy is an option of treatment for acute myocardial infarction and pulmonary embolism. Its use in both acute myocardial infarction and pulmonary embolisms has been described in guidelines [1, 2]. Unfortunately, thrombolytic therapy for renal artery thromboembolism causing renal artery infarcts has not been described in current guidelines. Acute myocardial infarction and pulmonary embolism thrombolysis protocols are applied in these patients. For this purpose, rt-PA (total of 100 mg followed by 15 mg intravenous fraction followed by 0.75 mg/kg (not exceeding 50 mg) in 30 minutes and then 0.50 mg/kg (with 35 mg not exceeded) infusion) is one of the most commonly used thrombolytic agents. In this application, rt-PA is given in 90 minutes and high dose [1, 2]. Unfortunately, with this protocol, 0.9–1.0% cerebral and 4–13% noncerebral hemorrhages are observed [1].

Unfortunately, there is no protocol developed to be applied in renal infarction due to the renal artery thromboembolism. According to these protocols, the administration times of this thrombolytic agent are below 90 minutes and the doses are very high. It means that the high rates of cerebral and noncerebral hemorrhages that develop due to both high doses and short treatment times are acceptable. Therefore, unlike the classic thrombolytic administration protocol, LDSITT protocol, which is thought to lower the risk of complications, was applied to this patient and complete dissolution of the thrombus was achieved without any complication.

LDSITT was first used by Özkan et al. [5] in patients with prosthetic valve thrombosis. They performed five different thrombolytic treatment strategies in patients with PVT. These regimens included rapid streptokinase (1.5 MU/3 h), slow streptokinase (1.5 MU/24 h), high-dose rt-PA (100 mg, 10 mg bolus, 90 mg/5 h), half-dose slow-infusion rt-PA (50 mg/6 h), and low-dose slow infusion rt-PA (25 mg/6 h). Treatment success did not differ between the groups. However, the complication rate was found to be significantly lower in the slow-infusion low-dose rt-PA group than in the other groups.

## 4. Conclusions

Traditional fibrinolytic treatment protocols that are used in the treatment of acute myocardial infarction and pulmonary embolism are associated with high complication rates in patients with RATE. Results of the few single-center, nonrandomized studies consisting of small patient groups showed that LDSITT is associated with low complication rates in patients with thromboembolism. According to our experience and very few literature reports, the LDSITT may be a treatment option in patients with RATE without any contraindication to thrombolytic therapy.

## Abbreviations

LDSITT: Low-dose slow-infusion thrombolytic therapy
LWFH: Low-molecular-weight heparin
PVT: Prosthetic valve thrombosis
RATE: Renal artery thromboembolism
rt-PA: Recombinant tissue plasminogen activator
UFH: Unfractionated heparin.

## Additional Points

*Aim*. We aimed to present a case of renal artery thromboembolism in which we applied low-dose slow-infusion thrombolytic therapy (LDSITT), and we have not observed any complication such as cerebral and noncerebral hemorrhage.

## Supplementary Materials

Supplementary 1 Video 1: first renal artery angiography at the left anterior oblique and cranial projection showing the multiple thromboembolic materials in the left renal artery branches.

Supplementary 2 Video 2: after second LDSITT, control renal artery angiography (right anterior oblique projection) showing residual thromboembolic materials in the left renal artery branches.

Supplementary 3 Video 3: after third LDSITT, final renal angiography (anteroposterior projection) showing normal left renal artery branches.

Supplementary 4 Video 4: another final renal angiography (right anterior oblique projection) showing normal left renal artery branches.
